# Deep Learning–Derived Retinal Age Detects Cognitive Impairment

**DOI:** 10.1016/j.xops.2026.101274

**Published:** 2026-06-04

**Authors:** Minkyu Kim, Kenneth Um, Gui-Shuang Ying, Benjamin J. Kim

**Affiliations:** 1 Department of Ophthalmology, Scheie Eye Institute, Perelman School of Medicine, University of Pennsylvania, Philadelphia, Pennsylvania; 2 Perelman School of Medicine at the University of Pennsylvania, Philadelphia, Pennsylvania; 3 Department of Ophthalmology, Center for Preventive Ophthalmology and Biostatistics, Scheie Eye Institute, University of Pennsylvania, Philadelphia, Pennsylvania

**Keywords:** Retinal age, Deep learning, Cognitive impairment, Fundus photography, Biomarker.

## Abstract

**Purpose:**

To investigate the association between deep learning–derived retinal age and cognitive function and to evaluate whether retinal age outperforms chronological age as a screening tool for cognitive impairment.

**Design:**

Cross-sectional analysis of the data from multicenter Artificial Intelligence–Ready and Exploratory Atlas for Diabetes Insights (AI-READI).

**Participants:**

We included 1049 participants (≥40 years) (mean [standard deviation] chronological age, 60.3 [11.2] years) enrolled in the AI-READI cohort, a multicenter, cross-sectional study.

**Methods:**

Retinal age was estimated from color fundus photographs using a pretrained deep learning model. Cognitive function was assessed using the Montreal Cognitive Assessment (MoCA), with cognitive impairment defined as a MoCA score <26. Generalized linear models were used to compare MoCA scores across retinal age quartiles. Poisson regression and logistic regression were used to evaluate the association between retinal age and cognitive impairment, adjusting for chronological age and cardiometabolic risk factors.

**Main Outcome Measures:**

Retinal age, MoCA scores, and cognitive impairment.

**Results:**

Retinal age correlated more strongly with MoCA total score than chronological age (R, –0.47 vs. –0.21). Retinal age was associated with cognitive impairment in a dose-response manner with adjusted risk ratio (RR) of 2.81, 6.03, and 10.13 for the second, third, and fourth quartiles in comparison to the first quartile of retinal age (all *P* < 0.001), while chronological age had a RR of 1.37, 1.42, and 1.65 for the second, third, and fourth quartiles compared with the first quartile (all *P* < 0.01). Retinal age had higher area under the curves (AUCs) than chronological age in detecting cognitive impairment (0.81 vs. 0.59 without covariates, and 0.83 vs. 0.66 with covariates). The combination of retinal age and chronological age yielded an AUC of 0.87. In the analysis stratified by diabetic severity, retinal age maintained a similar AUC (0.89 with covariates) for detecting cognitive impairment in nondiabetic healthy controls.

**Conclusions:**

Deep learning–derived retinal age is a biological biomarker that significantly outperforms chronological age in its association with cognitive function and has better performance in detecting cognitive impairment. This scalable, image-based biomarker has the potential for opportunistic screening within existing clinical workflows, facilitating earlier detection of cognitive decline.

**Financial Disclosure(s):**

Proprietary or commercial disclosure may be found in the Footnotes and Disclosures at the end of this article.

The rapidly aging population in the United States and globally has raised interest in scalable biomarkers that quantify biological aging and identify individuals at risk for age-related diseases. Though chronological age is a reliable indicator of these diseases, people of the same age differ markedly in health and functional status.^1^, ^2^, ^3^ This is due to fundamental biological heterogeneity in the rate of aging across individuals, a concept central to the geroscience framework. This framework posits that quantitative biomarkers of biological aging can capture multisystem decline better than chronological age.^4^, ^5^, ^6^ Early efforts to quantify biological aging, such as epigenetic clocks, proteomic and metabolomic signatures, and clinical indices, have been shown to predict age-related outcomes beyond chronological age, including cognitive function and mortality.^7^, ^8^, ^9^, ^10^ However, their cost and complexity have limited broad clinical development. These constraints have motivated the development of imaging-based biomarkers for aging.^11^^,^^12^

The ocular fundus offers a relatively low-cost, noninvasive view of neural and microvascular tissues owing to its shared embryological origin with the CNS. Leveraging these biological and practical advantages, recent work has used deep learning to estimate the image-derived retinal age from fundus photographs to evaluate its value as an aging biomarker. Previous studies demonstrated that deep learning–estimated retinal age is associated with a range of aging-related outcomes, such as morbidity, mortality, cardiometabolic, and neurological conditions.^13^, ^14^, ^15^ In a large cohort study, an artificial intelligence (AI)–derived retinal aging index demonstrated prognostic value for morbidity and mortality, even outperforming traditional biomarkers such as leukocyte telomere length and grip strength.^16^ In particular, Yu et al^17^ recently introduced an explainable deep learning approach to estimate retinal age from fundus photographs, and they found that higher retinal age was associated with increased risks of all-cause mortality and incident systemic disease beyond chronological age. Attention-map analyses indicated that the model prioritized vascular features, especially vessel fractal complexity and density, and higher retinal age corresponded to less complex vessel branching patterns and lower density.^17^ Taken together, these findings support the premise that retinal age captures systemic neurovascular and metabolic burden relevant to aging.

Although cognitive decline is a major consequence of aging and the retina is an accessible extension of the CNS, the relationship between retinal age and cognitive function remains largely underexplored.^18^ Some investigators have developed a deep learning classifier to determine mild cognitive impairment (MCI) from fundus photographs with moderate-to-high accuracy.^19^^,^^20^ For instance, a cross-sectional study developed a vision-ensemble model that achieved an area under the curve (AUC) of 0.77 for detecting MCI, defined by Montreal Cognitive Assessment (MoCA) score <26 or Mini Mental State Examination <27, in an external validation cohort of 196 patients with atrial fibrillation.^19^ Similarly, another cross-sectional study demonstrated a deep learning–based model trained to detect MCI using the same criteria, achieving an AUC of 0.73 in the external cohort of 601 patients with coronary artery disease.^20^ However, these binary classifiers, which only detect the presence or absence of MCI in a relatively small number of subjects, offer limited biological insight and fail to provide a continuous metric that captures the full spectrum of cognitive decline.

The recently introduced RetiPhenoAge is associated with cognitive decline.^21^ The model was designed by using fundus photographs to determine PhenoAge, a composite metric built from 9 blood biomarkers plus chronological age. Consequently, this model is strongly aligned with systemic inflammatory and metabolic status, rather than representing a purely image-based retinal aging construct.^21^ In contrast, the model by Yu et al^17^ provides an explainable, image-derived aging biomarker. The model was trained to predict the chronological age from healthy subjects, assuming that biological age corresponds to chronological age in healthy subjects. Therefore, in this study, we applied the model developed by Yu et al to derive the retinal age, examine its association with cognitive health, and evaluate its potential as a screening tool for cognitive impairment.

## Methods

### Study Setting and Design

We used data from the publicly available Artificial Intelligence–Ready and Exploratory Atlas for Diabetes Insights (AI-READI) cohort, a multicenter National Institutes of Health Bridge2AI project that collects cross-sectional multimodal health data.^22^ The cohort targets enrollment of 4000 adults aged ≥40 years, with recruitment stratified by self-identified race/ethnicity, diabetes status (healthy controls, prediabetes, diabetes), and sex. Participants were recruited at 3 academic sites (University of Washington, Seattle; University of Alabama at Birmingham; and University of California San Diego) during 2022 to 2026.^22^ All participants provided written informed consent, and the protocol was approved by the institutional review board at each site. This study adhered to the tenets of the Declaration of Helsinki. For this project, we used the publicly available data from 1067 participants as of October 31, 2025.

### Deep Learning Model for Retinal Age Estimation

Retinal age was estimated from color fundus photographs (5737 total images).^17^ The sample included 2796 images from the right eye and 2941 images from the left eye. The color fundus photographs used in this study were part of the AI-READI dataset, acquired using TOPCON Maestro2, Topcon Triton, and Optomed Aurora devices. The images from the Optomed Aurora were acquired in a nonmydriatic state, while images from the Topcon devices were collected under dilated conditions.^17^ We applied the pretrained deep learning model developed by Yu et al^17^ to calculate the retinal ages from the fundus photos collected from participants of the AI-READI study. Importantly, no AI-READI fundus images were used in the training, pretraining, or validation of the original model. This AI model process included the default automated image quality assessment. A total of 3323 images that failed the quality control step were deemed ungradable and were excluded from the analysis. The deep learning model estimated a retinal age for each fundus photograph. The mean retinal age from multiple photographs of a participant was calculated as the retinal age for each participant.^17^ The complete architectural, training, and preprocessing details of the model are described in the original publication.^17^

### Cognitive Assessment

Cognitive performance was assessed using the MoCA.^23^ The MoCA is a 30-point screening tool evaluating multiple cognitive domains, including short-term memory recall, visuospatial abilities, executive functioning, language, orientation, and attention/concentration. During the AI-READI on-site visit, trained research staff administered the test using a tablet-based electronic application. Scoring was conducted according to standard protocols. One point was added to the raw MoCA total score for participants with ≤12 years of education to adjust for educational level.^23^ Higher scores indicate better cognitive function.

### Statistical Analysis

We analyzed 1049 participants, excluding 18 with missing MoCA scores. Characteristics were compared across retinal age quartiles (Q1–Q4) using analysis of variance and chi-square tests. Pearson correlation coefficient was used for assessing correlation between MoCA scores and age (either retinal age, chronological age, or retinal age gap defined as retinal age minus chronological age). Generalized linear models compared MoCA scores across quartiles, with and without adjustment for risk factors including chronological age, years of education, body mass index, hemoglobin A1c (HbA1c), diabetes group, hypertension, kidney disease, hypercholesterolemia, circulation problems, and neurodegenerative disease.^24^ These same covariates were used across all multivariable models. Poisson regression models were used to evaluate the association between retinal age and cognitive impairment (defined as MoCA <26), and to calculate risk ratios (RRs) and their 95% confidence intervals (CIs). We calculated AUC from logistic regression to evaluate its discriminative ability. Comparison of AUCs from various models was performed using DeLong test.^25^ Area under the curve analysis was also stratified by diabetes status to further investigate the potential confounding effect of diabetic status. Variance inflation factors analysis was performed to check the multicollinearity among variables in the multivariable models. All analyses were conducted in Python 3.7.2 (Python Software Foundation), and 2-sided *P* < 0.05 was considered statistically significant.

## Results

### Participant Characteristics

The characteristics of the 1049 participants are reported in Table 1. The overall mean (standard deviation) chronological age was 60.3 (11.2) years, and the retinal age was 57.0 (9.4) years. Participants in the 4 retinal-age quartiles differed significantly in chronological age and several cardiometabolic profiles, including body mass index, HbA1c, hypertension, high blood cholesterol, and diabetes status (all *P* < 0.05), with older retinal age having higher rates of various comorbidities. Neurodegenerative disease was uncommon and did not differ significantly across retinal age quartiles (*P* = 0.08, Table 1). Notably, in contrast to chronological age which did not differ by diabetes severity (*P* = 0.16), retinal age progressively increased with greater diabetes severity (*P* < 0.01).

**Table 1:** Demographic and Clinical Characteristics of the Participants with Diabetes and Controls

Characteristics	All Participants (N = 1049)	Retinal Age Quartiles				*P* Value∗
		Q1 (N = 263)	Q2 (N = 262)	Q3 (N = 263)	Q4 (N = 261)	
Retinal age (yrs): mean (SD)	57.0 (9.4)	44.6 (5.1)	54.1 (2.0)	61.0 (1.8)	68.4 (3.3)	<0.01
Chronological age (yrs): mean (SD)	60.3 (11.2)	49.3 (8.0)	59.2 (9.2)	62.8 (7.8)	70.0 (8.1)	<0.01
Education level, n(%)						0.09
High school or below (≤12)	41 (3.9%)	7 (2.7%)	7 (2.7%)	17 (6.5%)	10 (3.8%)	
College (13–16)	594 (56.6%)	134 (51.0%)	159 (60.7%)	148 (56.3%)	153 (58.6%)	
Graduate school (17–25)	402 (38.3%)	118 (44.9%)	92 (35.1%)	97 (36.9%)	95 (36.4%)	
Unknown	12 (1.1%)	4 (1.5%)	4 (1.5%)	1 (0.4%)	3 (1.1%)	
BMI (kg/m^2^): mean (SD)	30.7 (7.7)	31.9 (9.0)	30.8 (7.4)	30.7 (8.0)	29.32 (6.0)	<0.01
HbA1c%, mean (SD)	6.1 (1.2)	5.9 (1.3)	6.0 (1.1)	6.2 (1.4)	6.25 (1.0)	<0.01
CRP (mg/L): mean (SD)	3.8 (7.4)	4.5 (9.0)	3.3 (6.0)	3.9 (6.0)	3.5 (7.9)	0.27
Hypertension, yes(%)	562 (53.6%)	113 (43.0%)	143 (54.6%)	155 (58.9%)	151 (57.9%)	<0.01
Kidney problems, yes(%)	116 (11.1%)	19 (7.2%)	31 (11.8%)	29 (11.0%)	37 (14.2%)	0.08
High blood cholesterol, yes(%)	537 (51.2%)	103 (39.2%)	133 (50.8%)	150 (57.0%)	151 (57.9%)	<0.01
Circulation problem, yes(%)	84 (8.0%)	13 (4.9%)	19 (7.3%)	22 (8.4%)	30 (11.5%)	0.05
Diabetes group						
Healthy control	367 (35.0%)	113 (43.0%)	87 (33.2%)	89 (33.8%)	78 (29.9%)	0.01
Prediabetes	237 (22.6%)	57 (21.7%)	71 (27.1%)	50 (19.0%)	59 (22.6%)	0.16
Medication-controlled diabetes	318 (30.3%)	67 (25.5%)	81 (30.9%)	88 (33.5%)	82 (31.4%)	0.23
Insulin-dependent diabetes	127 (12.1%)	26 (9.9%)	23 (8.8%)	36 (13.7%)	42 (16.1%)	0.04
Neurodegenerative disease, yes(%)	42 (4.0%)	7 (2.7%)	7 (2.7%)	11 (4.2%)	17 (6.5%)	0.08
Alzheimer disease	1 (0.1%)	0 (0.0%)	0 (0.0%)	0 (0.0%)	1 (0.4%)	0.39
Mild cognitive impairment	30 (2.9%)	7 (2.7%)	3 (1.1%)	8 (3.0%)	12 (4.6%)	0.13
Parkinson disease	6 (0.6%)	0 (0.0%)	2 (0.8%)	2 (0.8%)	2 (0.8%)	0.57
Multiple sclerosis	6 (0.6%)	0 (0.0%)	2 (0.8%)	1 (0.4%)	3 (1.1%)	0.34

BMI = body mass index; CRP = C-reactive protein; HbA1c = hemoglobin A1c; SD = standard deviation.

∗ *P* values were calculated using 1-way analysis of variance for continuous measures and chi-square test for categorical measure.

### Correlations of MoCA Scores with Chronological Age and Retinal Age

**Figure 1:**
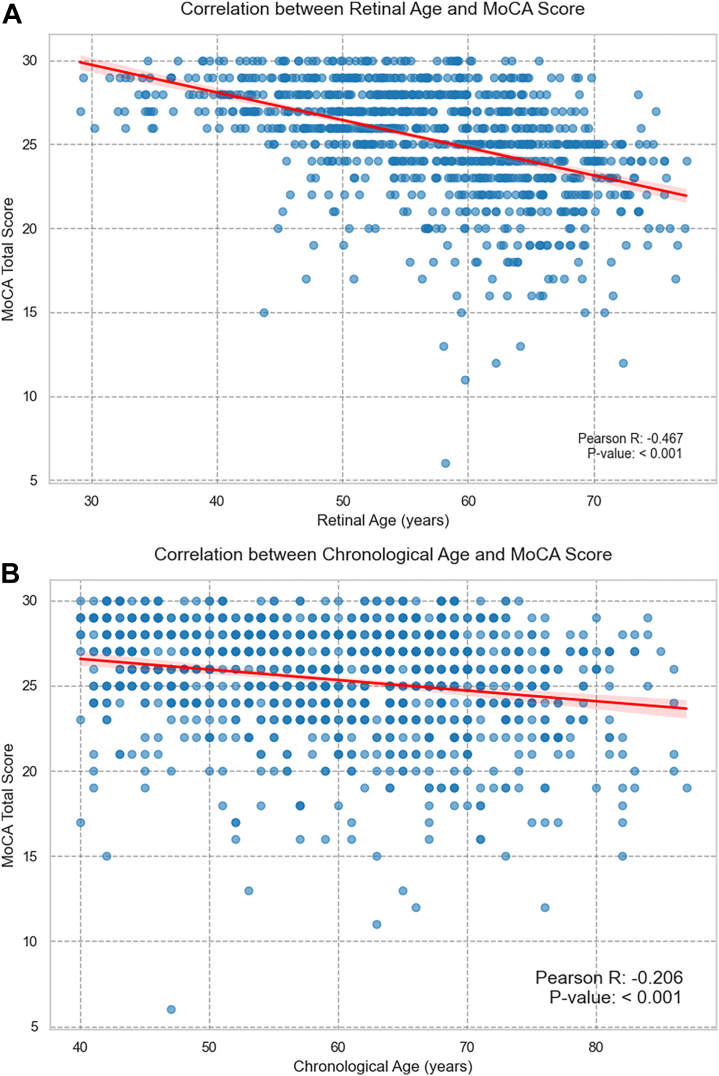
**A,** Scatter plot for retinal age and total MoCA score. **B,** Scatter plot for chronological age and total MoCA score. MoCA = Montreal Cognitive Assessment.

Retinal age was moderately correlated with chronological age (R = 0.69) but showed consistently stronger negative correlations with MoCA scores than chronological age and retinal age gap (Table 2, Fig 1). For MoCA total score, the correlation was R = –0.47 for retinal age versus R = –0.21 for chronological age, and R = –0.25 for retinal age gap. Retinal age correlations were stronger than chronological age for all domains, including Combined Memory Index Score (R = –0.32 vs. R = –0.20) and Abstraction (R = –0.11 vs. R = –0.002).

**Table 2:** Correlation of Montreal Cognitive Assessment Scores with Chronological Age and Retinal Age

Domain	Chronological Age		Retinal Age		Retinal Age Gap†	
	Pearson Correlation Coefficient	*P* Value	Pearson Correlation Coefficient	*P* Value	Pearson Correlation Coefficient	*P* Value
Abstraction (0–2)	–0.002	0.95	–0.11	<0.01	–0.13	<0.01
Abstraction time (sec)	0.05	0.10	0.12	<0.01	0.07	0.02
Orientation (0–6)	–0.11	<0.01	–0.16	<0.01	–0.03	0.32
Orientation time (sec)	0.14	<0.01	0.20	<0.01	0.03	0.40
CMIS score (0–15)∗	–0.20	<0.01	–0.32	<0.01	–0.09	<0.01
Total score (0–30)	–0.21	<0.01	–0.47	<0.01	–0.25	<0.01

CMIS = Combined Memory Index Score.

∗ Combined Memory Index Score is defined by combined score of Montreal Cognitive Assessment delayed recall with no cue, Montreal Cognitive Assessment delayed recall category cue (not included in total score), Montreal Cognitive Assessment delayed recall multiple choice cue (not included in total score).

† Retinal age gap is calculated as retinal age minus chronological age.

### Differences in MoCA Scores across Quartiles of Retinal Age and Chronological Age

Montreal Cognitive Assessment total scores differed significantly across retinal age quartiles in both unadjusted and adjusted analyses, showing a gradual decline from Q1 to Q4 with adjusted mean MoCA scores of 27.7, 26.5, 24.6, and 23.2 for Q1 to Q4 of retinal age, respectively (*P* < 0.001, Table 3). At the domain level, scores for the Abstraction and Combined Memory Index Score domains showed a significant reduction across quartiles (both *P* < 0.001). Abstraction Time and Orientation Time were also significantly longer in the higher retinal age quartiles (all *P* < 0.05). In contrast, Orientation did not differ significantly after adjustment (*P* = 0.17). For chronological age quartiles, MoCA total scores also differed significantly after adjustment (*P* < 0.001), but did not show a clear gradual decline, with adjusted mean MoCA total scores of 26.5, 25.6, 25.4, and 24.6 for Q1 to Q4 of chronological age, respectively (Table S1, available at www.ophthalmologyscience.org). Significant reductions were also observed for the Combined Memory Index Score Score (*P* < 0.001) and Orientation (*P* < 0.05), while Abstraction Time and Orientation Time remained longer at older chronological age (*P* < 0.01). Only the Abstraction domain was not significant (*P* = 0.76). However, decline of these domain-level scores over 4 quartiles were generally smaller than those observed for retinal age (Table S1).

**Table 3:** Univariate and Multivariate Analysis for Comparison of Montreal Cognitive Assessment Scores across Retinal Age Groups

Domain†	Analysis	Mean (SE)				*P* Value
		Retinal Age Q1 (N = 263)	Retinal Age Q2 (N = 262)	Retinal Age Q3 (N = 263)	Retinal Age Q4 (N = 261)	
Abstraction (0–2)	Unadjusted	1.92 (0.02)	1.90 (0.02)	1.79 (0.03)	1.81 (0.03)	<0.001
	Adjusted∗	1.96 (0.03)	1.91 (0.03)	1.80 (0.03)	1.78 (0.03)	<0.001
Abstraction time (sec)	Unadjusted	39.93 (1.22)	43.44 (1.98)	43.00 (1.95)	50.43 (2.78)	<0.001
	Adjusted∗	40.06 (2.86)	43.82 (2.53)	42.75 (2.58)	50.70 (2.85)	<0.01
CMIS score (0–15)‡	Unadjusted	13.44 (0.15)	12.53 (0.18)	11.88 (0.19)	11.02 (0.20)	<0.001
	Adjusted∗	13.42 (0.25)	12.68 (0.23)	12.06 (0.23)	11.33 (0.25)	<0.001
Orientation (0–6)	Unadjusted	5.98 (0.01)	5.93 (0.02)	5.89 (0.02)	5.85 (0.02)	<0.001
	Adjusted∗	5.96 (0.03)	5.94 (0.02)	5.90 (0.02)	5.88 (0.03)	0.17
Orientation time (sec)	Unadjusted	26.36 (0.57)	29.92 (0.79)	30.55 (0.88)	32.97 (0.82)	<0.001
	Adjusted∗	27.01 (1.08)	29.89 (0.96)	29.98 (0.97)	31.60 (1.07)	<0.05
Total score (0–30)	Unadjusted	27.16 (0.14)	26.19 (0.17)	24.41 (0.23)	23.18 (0.19)	<0.001
	Adjusted∗	27.71 (0.25)	26.49 (0.22)	24.60 (0.23)	23.18 (0.25)	<0.001

CMIS = Combined Memory Index Score; SE = standard error.

∗ Adjusted by age, education, body mass index, hemoglobin A1c, diabetes groups, hypertension, kidney problems, high blood cholesterol, circulation problems, and neurodegenerative diseases. Subjects (n = 44) with missing data in any of these covariates were excluded from multivariable analysis.

† Higher values indicate better performance for the Abstraction, the CMIS, the Orientation, and the Montreal Cognitive Assessment (MoCA) total score. Conversely, shorter times indicate better performance on the Abstraction Time and Orientation Time, which index processing speed and are not included in the MoCA total score.

‡ Combined Memory Index Score is defined by the combined score of MoCA delayed recall with no cue, MoCA delayed recall category cue (not included in total score), and MoCA delayed recall multiple choice cue (not included in total score).

### Risk of Cognitive Impairment

Among 1049 participants, 492 (46.9%) had cognitive impairment, defined as MoCA score <26. Of these, 462 (93.9%) were classified as mild (MoCA 18–25), 29 (5.9%) as moderate (MoCA 10–17), and 1 (0.2%) as severe (MoCA <10). The prevalence rate of cognitive impairment increased with retinal age quartiles, with 14.4%, 31.3%, 60.5%, and 81.6% in retinal age Q1, Q2, Q3, and Q4, respectively. In multivariable analysis, the adjusted RRs were 2.81 (95% CI, 2.00–3.96) for Q2, 6.03 (95% CI, 4.41–8.23) for Q3, and 10.13 (95% CI, 7.32–14.03) for Q4 compared with Q1 (all *P* < 0.001, Table 4).

**Table 4:** Univariable and Multivariable Analysis for Association between Retinal Age, Chronological Age, and Cognitive Impairment

	Univariate Analysis				Multivariate Analysis†			
	n	Cognitive Impairment∗ n (%)	Risk Ratio (95% CI)	*P* Value	n	Cognitive Impairment∗ n (%)	Adjusted Risk Ratio (95% CI)	*P* Value
Retinal age								
Q1	263	38 (14.4%)	Reference		256	37 (14.5%)	Reference	
Q2	262	82 (31.3%)	2.17 (1.53-3.06)	<0.001	253	78 (30.8%)	2.81 (2.00-3.96)	<0.001
Q3	263	159 (60.5%)	4.18 (3.07-5.70)	<0.001	253	152 (60.1%)	6.03 (4.41-8.23)	<0.001
Q4	261	213 (81.6%)	5.65 (4.19-7.62)	<0.001	243	196 (80.7%)	10.13 (7.32-14.03)	<0.001
Chronological age								
Q1	263	91 (34.6%)	Reference		255	88 (34.5%)	Reference	
Q2	287	142 (49.5%)	1.43 (1.17-1.75)	<0.01	275	131 (47.6%)	1.37 (1.11-1.68)	<0.01
Q3	267	128 (47.9%)	1.39 (1.13-1.71)	<0.01	261	125 (47.9%)	1.42 (1.15-1.76)	<0.01
Q4	232	131 (56.5%)	1.63 (1.33-2.00)	<0.01	214	119 (55.6%)	1.65 (1.34-2.04)	<0.01

CI = confidence interval; MoCA = Montreal Cognitive Assessment.

∗ MoCA total score <26.

† From multivariable Poisson regression model with adjusted by age, education, body mass index, hemoglobin A1c, diabetes groups, hypertension, kidney problems, high blood cholesterol, circulation problems, and neurodegenerative diseases. Subjects (n = 44) with missing data in any of these covariates were excluded from multivariable analysis.

The risk difference of cognitive impairment across chronological age quartiles was considerably smaller, with prevalence rates of 34.6%, 49.5%, 47.9%, and 56.5% for Q1, Q2, Q3, and Q4, respectively. In the multivariable analysis, the adjusted RRs relative to Q1 were 1.37 (95% CI, 1.11–1.68) for Q2, 1.42 (95% CI, 1.15–1.76) for Q3, and 1.65 (95% CI, 1.34–2.04) for Q4 (all *P* < 0.01, Table 4). A sensitivity analysis using 5-year retinal age intervals confirmed a consistent dose-response relationship (Table S2, available at www.ophthalmologyscience.org). Also, another sensitivity analysis excluding 21 participants with best-corrected visual acuity worse than 20/40 and 9 participants without best-corrected visual acuity data showed consistent results, with adjusted RR for cognitive impairment remaining robust across retinal age quartiles (Table S3, available at www.ophthalmologyscience.org).

### Detection of Cognitive Impairment

**Figure 2:**
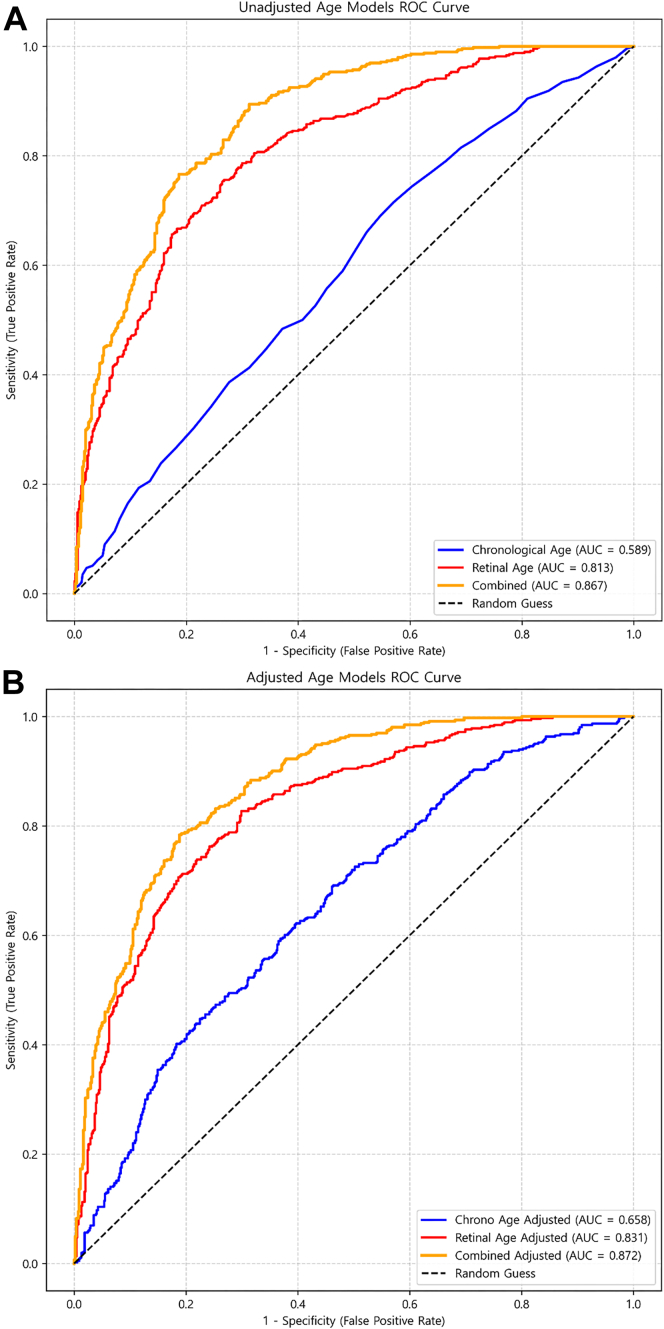
**A,** Unadjusted model of ROC curve for predicting cognitive impairment using retinal age, chronological age or combination. **B,** Adjusted model of ROC curve for predicting cognitive impairment using retinal age, chronological age, or combination with other covariates. AUC = area under the curve; ROC = receiver operating characteristic.

In univariate models, retinal age showed better performance in detecting cognitive impairment than chronological age, with an AUC of 0.81 (95% CI, 0.79–0.84) for retinal age and 0.59 (95% CI, 0.56–0.62) for chronological age. Combining chronological age and retinal age achieved an AUC of 0.87 (95% CI, 0.84–0.89) (Table 5, Fig 2A).

**Table 5:** Area Under ROC Curve for the Prediction of Cognitive Impairment from Various Models Using Retinal Age or Chronological Age and Stratified by Diabetic Status

Predictors	Area Under ROC Curve (95% CI)	
	Without Covariates	With Covariates∗
All participants		
Chronological age	0.59 (0.56–0.62)	0.66 (0.63–0.70)
Retinal age	0.81 (0.79–0.84)	0.83 (0.81–0.86)
Chronological age + retinal age	0.87 (0.84–0.89)	0.87 (0.85–0.90)
Retinal age model stratified by diabetic status		
Healthy control group	0.84 (0.80–0.87)	0.89 (0.86–0.93)
Prediabetic group	0.80 (0.74–0.85)	0.88 (0.84–0.93)
Diabetic group†	0.79 (0.75–0.84)	0.84 (0.81–0.89)

CI = confidence interval; ROC = receiver operating characteristic.

∗ Covariates included education, body mass index, hemoglobin A1c, diabetes groups, hypertension, kidney problems, high blood cholesterol, circulation problems, and neurodegenerative diseases.

† Includes participants using oral medication, noninsulin injectables, or insulin-dependent.

When including other risk factors of cognitive function (chronological age, years of education, body mass index, HbA1c, diabetes group, hypertension, kidney disease, hypercholesterolemia, circulation problems, and neurodegenerative disease), AUCs improved to 0.83 (95% CI, 0.81–0.86) for retinal age, 0.66 (95% CI, 0.63–0.70) for chronological age, and 0.87 (95% CI, 0.85–0.90) for the combination of chronological age and retinal age (Table 5, Fig 2B).

To further investigate the robustness of retinal age's ability to detect cognitive impairment independent of diabetes status, we performed an AUC analysis stratified by diabetes status. Retinal age detected cognitive impairment similarly well with AUCs of 0.84 (95% CI: 0.80–0.87) in healthy controls, 0.80 (95% CI: 0.74–0.85) in prediabetes group, and 0.79 (0.75–0.84) in medication or insulin-dependent diabetes group. When the other risk factors of cognitive function were included, the AUC was 0.89 (95% CI, 0.86–0.93) in healthy controls, 0.88 (95% CI, 0.84–0.93) in prediabetes group, and 0.84 (95% CI, 0.81–0.89) in medication or insulin-dependent diabetes group (Table 5).

## Discussion

We found that deep learning–derived retinal age outperformed chronological age in its association with MoCA performance and detection of cognitive impairment. While prior studies used a cross-sectional data set of retinal images to train their model,^19^, ^20^, ^21^^,^^26^ our study utilized a state-of-the-art model^17^ that incorporates cross-sectional and longitudinal pretraining (to model individual dynamic aging changes) and label distribution learning (to calculate a more precise age estimation). We have applied this advanced biomarker to a large, diverse, multicenter US cohort, specifically evaluating cognitive impairment and demonstrating robust utility. Early cognitive impairment detection is critical for managing reversible causes and initiating disease-modifying therapies; therefore, a scalable, accessible screening tool like retinal age holds significant potential.^27^, ^28^, ^29^

The retinal age estimated from the deep learning model of fundus photographs^17^ demonstrated strong discriminatory performance, achieving an AUC of 0.81 for classifying cognitive impairment, which was markedly higher than that of chronological age (AUC 0.59) (Table 5). This performance compares favorably with other recent deep learning classifiers for cognitive impairment. For instance, one study involving 196 patients with atrial fibrillation developed a vision-ensemble model using fundus photographs, achieving an AUC of 0.77 in detecting cognitive impairment in its external test set.^19^ Similarly, another study demonstrated an AI model using fundus photographs that achieved an AUC of 0.73 in detecting MCI in an external set of 601 patients with coronary artery disease.^20^

In our data set, chronological age was similar across the diabetes groups (*P* = 0.16), whereas retinal age increased with diabetes severity (*P* < 0.01), suggesting that retinal age provides information about the biological aging process. Even after adjusting for demographics, comorbidities, diabetes severity group, and HbA1c, retinal age retained significant associations with cognitive function (Table 3) and demonstrated a strong ability to discriminate cognitive impairment (Table 4). The data showed substantial discriminative power over chronological age (AUC 0.81 vs. 0.59) (Table 5). Notably, the combination of retinal age and chronological age together provided an AUC of 0.87 (Table 5), and the addition of other risk factors of cognitive impairment into the model did not improve the AUC further (AUC 0.87), indicating that much of the covariate-related signal was already captured by the 2 age metrics. It highlights the potential of retinal age to serve as a more precise screening tool that could replace or significantly augment traditional age-based risk assessments in clinical settings where chronological age is already a known baseline factor or single factor. Given that diabetes is a major risk factor for both retinal microvascular change and cognitive decline, there is a critical potential for confounding.^30^ To further test the robustness of retinal age independent of diabetes status, we performed a subgroup analysis stratified by diabetes status (Table 5). We found that the AUC remained high even within the healthy control group (AUC 0.84), and it showed a similar strong performance in the prediabetes group (AUC 0.80) and the diabetes group (AUC 0.79). These findings suggest retinal age provides an independent signal for cognitive status, unconfounded by diabetes, supporting its use as an image-only screening biomarker. Additionally, sensitivity analysis excluding participants with visual acuity worse than 20/40 yielded consistent results (Table S3), suggesting visual impairment should not confound our findings.

The recently proposed RetiPhenoAge is associated with cognitive decline and incident dementia.^21^ However, the model was trained to predict PhenoAge, a composite metric of 9 blood biomarkers plus chronological age. Thus, the model demonstrates that retinal images can capture a signal strongly aligned with systemic inflammatory and metabolic status.^21^ Nonetheless, this reliance on a laboratory-derived ground truth makes it difficult to isolate the direct association between intrinsic retinal aging and cognitive health. This highlights a specific gap regarding whether a retinal aging biomarker, independent of a laboratory-derived target, can also predict cognitive status to demonstrate the direct association between retinal aging and cognitive health. To address this gap and explore the potential of a purely image-derived aging signal, we selected the recent AI model developed by Yu et al.^17^ We note that the study by Yu et al^17^ did not report an association between retinal age and incident dementia. However, their study used a broad definition of dementia that encompassed a range of neurodegenerative conditions, whereas our more specific analysis centered on the MoCA-based cognitive decline and MoCA-defined cognitive impairment, which carries a high risk of progressing to incident dementia.^31^ Furthermore, Yu et al did see a significant association of retinal age with incident dementia with a sensitivity analysis involving more detailed age adjustment (reported in their study's Supplementary file).^17^

We used an explainable, image-derived aging biomarker that incorporates snapshot and longitudinal self-supervised pretraining to capture dynamic retinal changes that reflect the biological aging process. The model focuses on specific retinal microvascular features, including vessel fractal complexity and density for estimating retinal age, providing its explainability. However, this explainability evidence is external and was validated in the original article.^17^ The biological plausibility of our findings is supported by links between these retinal features and cerebral small-vessel disease, a known substrate of cognitive decline.^5^^,^^20^^,^^32^, ^33^, ^34^ Thus, the model provides an interpretable biomarker reflecting plausible mechanisms of cerebrovascular health and neurodegenerative risk.

Beyond high AUC performance, the practical advantage is also important to consider. Retinal age can be acquired in minutes by using widely available nonmydriatic cameras. It can be integrated naturally into existing diabetic retinopathy screening and teleophthalmology workflows. The model pipeline integrates an automated quality control process, ensuring standardized and reliable retinal age estimation while streamlining workflows without requiring manual review. Despite acquisition heterogeneity across 3 camera systems and both mydriatic and nonmydriatic conditions, retinal age demonstrated consistently strong associations with cognitive outcomes. Given the MoCA's well-established sensitivity (reported as 93.7%)^35^ for detecting MCI (MoCA <26), this retinal age from fundus photographs presents a scalable opportunity for screening, potentially facilitating screening and intervention at the stage of early cognitive decline, rather than moderate impairment or dementia.^35^

Our study has several limitations. First, our findings are from the AI-READI cohort, which is enriched for diabetes—a major risk factor for both retinal microvascular change and cognitive decline.^30^ Although our multivariable models adjusted for diabetes status retained a strong independent association (Table 4), and subgroup analyses stratified by diabetes status showed consistent performance across healthy, prediabetic, and diabetic groups (Table 5), the cohort's diabetes enrichment may still limit generalizability to nondiabetic populations. Second, as our study applied a pretrained model as a fixed inference tool rather than further calibrating and evaluating deep learning model performance, the degree to which model performance generalizes in this data set remains dependent on the robustness established during the original model development.^17^ Moreover, deep learning–derived retinal age carries inherent prediction uncertainty and potential age-dependent bias. However, random measurement error in the predictor would attenuate associations toward the null through regression dilution bias and reduce discriminative performance, suggesting that our reported effect sizes and AUC values are likely conservative estimates. Third, the cross-sectional design limits inferring causality. Longitudinal studies are needed to evaluate the prognostic utility for incident cognitive decline. Fourth, associations may be subject to residual confounding from unmeasured variables, including sociodemographic factors, language, genetic predispositions (e.g., apolipoprotein E ε4 status), and medications. Additionally, cognitive impairment was defined solely by MoCA screening rather than comprehensive clinical diagnosis, which may introduce misclassification bias. Finally, our analysis compared retinal age only against chronological age, not other emerging retinal biomarkers or multimodal deep learning–derived biomarkers, which remains a direction for future research.

## Conclusion

In conclusion, this study demonstrates that a deep learning–derived retinal age from fundus photographs is a biologically explainable biomarker that significantly outperforms chronological age in correlating with cognitive function and for detecting cognitive impairment. We tested this biomarker with the large and diverse AI-READI cohort, establishing its robustness against key systemic confounders like diabetes. The retinal age's high discriminative power for cognitive impairment, achieved using fundus photographs alone and supported by integrated automated quality control, positions it as a practical, generalizable, and scalable tool that could be used within existing clinical workflows.
