# Supplementary material for: Deep Learning–Derived Retinal Age Detects Cognitive Impairment
Source: Ophthalmol Sci. 2026 Jun 4;6(8):101274. doi: 10.1016/j.xops.2026.101274 (PMC13355701; doi:10.1016/j.xops.2026.101274)
Supplement: Table S1 [file mmc1.pdf]

Table S1: Univariate and multivariate analysis for comparison for association between Montreal Cognitive Assessment (MoCA) scores across Chronological age groups

		Mean (SE)				
**Domain****	**Analysis**	Chronological Age Q1 (N=247)	Chronological Age Q2 (N=247)	Chronological Age Q3 (N=246)	Chronological Age Q4 (N=247)	**P-value**
**Abstraction (0-2)**	Unadjusted	1.87 (0.02)	1.82 (0.03)	1.88 (0.03)	1.85 (0.03)	0.36
	Adjusted*	1.87 (0.03)	1.84 (0.03)	1.87 (0.03)	1.86 (0.03)	0.76
**Abstraction Time (seconds)**	Unadjusted	41.99 (1.28)	46.46 (2.05)	39.28 (1.04)	49.73 (3.48)	<0.01
	Adjusted*	41.76 (2.31)	45.78 (2.45)	40.19 (2.63)	49.72 (2.88)	<0.01
**CMIS Score (0-15)*****	Unadjusted	12.88 (0.17)	12.44 (0.18)	12.24 (0.18)	11.17 (0.22)	<0.001
	Adjusted*	13.10 (0.21)	12.72 (0.22)	12.43 (0.24)	11.32 (0.26)	<0.001
**Orientation (0-6)**	Unadjusted	5.96 (0.01)	5.93 (0.02)	5.90 (0.02)	5.85 (0.03)	<0.01
	Adjusted*	5.95 (0.02)	5.94 (0.02)	5.91 (0.02)	5.87 (0.03)	<0.05
**Orientation Time (seconds)**	Unadjusted	27.79 (0.76)	30.05 (0.75)	29.82 (0.76)	32.49 (0.85)	<0.001
	Adjusted*	27.54 (0.88)	29.36 (0.93)	29.47 (1.00)	31.79 (1.09)	<0.01
**Total Score (0-30)**	Unadjusted	26.23 (0.19)	25.16 (0.19)	25.21 (0.22)	24.25 (0.22)	<0.001
	Adjusted*	26.48 (0.22)	25.57 (0.24)	25.44 (0.26)	24.59 (0.28)	<0.001

*Adjusted by age, education, BMI, HbA1c, diabetes groups, hypertension, kidney problems, high blood cholesterol, circulation problems, and neurodegenerative diseases. Subjects (n=44) with missing data in any of these covariates were excluded from multivariable analysis.
** Higher values indicate better performance for the Abstraction, the Combined Memory Index Score (CMIS), the Orientation, and the MoCA Total Score. Conversely, shorter times indicate better performance on the Abstraction Time and Orientation Time, which index processing speed and are not included in the MoCA Total Score.
***CMIS(Combined Memory Index Score) is defined by combined score of MOCA delayed recall with no cue, MOCA delayed recall category cue (not included in total score), MOCA delayed recall multiple choice cue (not included in total score
