# Supplementary material for: Deep Learning–Derived Retinal Age Detects Cognitive Impairment
Source: Ophthalmol Sci. 2026 Jun 4;6(8):101274. doi: 10.1016/j.xops.2026.101274 (PMC13355701; doi:10.1016/j.xops.2026.101274)
Supplement: Table S2 [file mmc2.pdf]

Table S2. Univariable and multivariable analysis for association between 5-year retinal age groups and cognitive impairment.

	Univariate Analysis				Multivariate Analysis **			
**Retinal Age (years)**	**n**	**Cognitive Impairment* n(%)**	**Risk Ratio (95% CI)**	**P-value**	**n**	**Cognitive Impairment* n(%)**	**Adjusted Risk Ratio (95% CI)**	**P-value**
< 50 (ref)	242	32 (13.2)	Reference	—	235	31 (13.2)	Reference	—
50–54	192	51 (26.6)	2.01 (1.35–2.99)	<0.001	185	48 (25.9)	2.51 (1.69–3.74)	<0.001
55–59	191	85 (44.5)	3.37 (2.35–4.82)	<0.001	184	81 (44.0)	4.74 (3.31–6.80)	<0.001
60–64	205	140 (68.3)	5.16 (3.69–7.23)	<0.001	197	134 (68.0)	8.19 (5.79–11.58)	<0.001
≥65	219	184 (84.0)	6.35 (4.58–8.82)	<0.001	204	169 (82.8)	12.27 (8.54–17.63)	<0.001

*Adjusted for chronological age, years of education, BMI, HbA1c, diabetes group, hypertension, kidney problems, high blood cholesterol, circulation problems, and neurodegenerative disease. Subjects (n=44) with missing data in any of these covariates were excluded from multivariable analysis.
