# Supplementary material for: Deep Learning–Derived Retinal Age Detects Cognitive Impairment
Source: Ophthalmol Sci. 2026 Jun 4;6(8):101274. doi: 10.1016/j.xops.2026.101274 (PMC13355701; doi:10.1016/j.xops.2026.101274)
Supplement: Table S3 [file mmc3.pdf]

Table S3. Univariable and multivariable analysis for association between retinal age, chronological age and cognitive impairment among participants with best corrected visual acuity 20/40 or better

	Univariate Analysis				Multivariate Analysis **			
	n	Cognitive Impairment* n(%)	Risk Ratio (95% CI)	P-value	n	Cognitive Impairment* n(%)	Adjusted Risk Ratio (95% CI)	P-value
**Retinal age**								
Q1	255	35 (13.7%)	Reference		244	33 (13.5%)	Reference	
Q2	255	83 (32.5%)	2.37 (1.66-3.38)	<0.001	244	79 (32.4%)	3.04 (2.13-4.34)	<0.001
Q3	255	146 (57.3%)	4.17 (3.01-5.78)	<0.001	244	138 (56.6%)	5.95 (4.26-8.32)	<0.001
Q4	254	207 (81.5%)	5.94 (4.34-8.12)	<0.001	240	193 (80.4%)	10.59 (7.48-14.99)	<0.001
**Chronological age**								
Q1	255	88 (34.5%)	Reference		245	85 (34.7%)	Reference	
Q2	255	122 (47.8%)	1.39 (1.12-1.71)	<0.01	241	110 (45.6%)	1.32 (1.06-1.64)	<0.01
Q3	255	120 (47.1%)	1.36 (1.10-1.69)	<0.01	248	117 (47.2%)	1.40 (1.13-1.74)	<0.01
Q4	254	141 (55.5%)	1.61 (1.31-1.97)	<0.01	238	131 (55.0%)	1.64 (1.33-2.03)	<0.01

*MoCA total score <26

**From multivariable Poisson regression model with adjusted by age, education, BMI, HbA1c, diabetes groups, hypertension, kidney problems, high blood cholesterol, circulation problems, and neurodegenerative diseases. Subjects (n=44) with missing data in any of these covariates were excluded from multivariable analysis
